# Long Non-Coding RNAs and Metabolic Rewiring in Pancreatic Cancer

**DOI:** 10.3390/cancers15133486

**Published:** 2023-07-04

**Authors:** Bruna Dalmasso, Paola Ghiorzo

**Affiliations:** 1 IRCCS Ospedale Policlinico San Martino, Genetics of Rare Cancers, 16132 Genoa, Italy; paola.ghiorzo@unige.it; 2 Department of Internal Medicine and Medical Specialties, University of Genoa, 16132 Genoa, Italy

**Keywords:** long non-coding RNA, pancreatic cancer, PDAC, hypoxia, autophagy, metabolic rewiring, chemoresistance, therapeutic targets

## Abstract

**Simple Summary:**

Altered metabolism is one of the main driving forces of pancreatic cancer development, progression, and response to treatment. Long non-coding RNAs, which regulate multiple cellular functions, are frequently aberrantly expressed in pancreatic cancer. As lncRNAs can be measured in tissue and plasma, and can be silenced by different mechanisms, there is growing interest in their potential as biomarkers and/or therapeutic targets. Considering that several lncRNAs are implicated in metabolic homeostasis, this review focuses on the impact of lncRNA disruption in pancreatic cancer metabolic rewiring.

**Abstract:**

Pancreatic adenocarcinoma is a highly aggressive disease with a poor prognosis. The reprogramming of energetic metabolism has long been implicated in pancreatic tumorigenesis and/or resistance to treatment. Considering that long non-coding RNA dysregulation has been described both in cancerogenesis and in the altered homeostasis of several metabolic pathways, metabolism-associated lncRNAs can contribute to pancreatic cancer evolution. The objective of this review is to assess the burden of lncRNA dysregulation in pancreatic cancer metabolic reprogramming, and its effect on this tumor’s natural course and response to treatment. Therefore, we reviewed the available literature to assess whether metabolism-associated lncRNAs have been found to be differentially expressed in pancreatic cancer, as well as whether experimental evidence of their role in such pathways can be demonstrated. Specifically, we provide a comprehensive overview of lncRNAs that are implicated in hypoxia-related pathways, as well as in the reprogramming of autophagy, lipid metabolism, and amino acid metabolism. Our review gathers background material for further research on possible applications of metabolism-associated lncRNAs as diagnostic/prognostic biomarkers and/or as potential therapeutic targets in pancreatic adenocarcinoma.

## 1. Introduction

### 1.1. Epidemiology and Treatment

Pancreatic cancer is the seventh leading cause of death by cancer worldwide, with a dismal prognosis, as the number of deaths registered are almost as many as the number of diagnoses. Its incidence is higher in countries with an elevated Human Development Index (HDI) and in the next few years it is projected to become the third and the second leading cause of cancer death in Europe in the United States, respectively [1,2].

Pancreatic adenocarcinoma (PDAC), which comprises the majority of newly diagnosed pancreatic cancers, is usually unresectable at diagnosis. However, the prognosis is also dismal for patients diagnosed at an early stage, as the five-year survival rate in this small subset does not exceed 20% [3].

Therapeutic options for locally advanced and metastatic disease include platinum-based chemotherapy regimens, as well as gemcitabine and nab-paclitaxel. Considering that, however, chemoresistance develops eventually, novel agents targeted to cancer cell-specific vulnerabilities are being studied, such as the introduction of PARP-inhibitors for tumors harboring *BRCA1/2* pathogenic variants [4,5]. Several mechanisms of cancer development and progression are being studied for their actionability. Among them, there is a growing body of literature exploring the metabolic pathways involved in cancer cell survival, since reprogrammed cell metabolism is one of the hallmarks of cancer [6].

### 1.2. Metabolic Rewiring in Pancreatic Cancer

PDAC is a heavily metabolic rewired tumor, and multiple metabolic pathways have long been implicated in PDAC development and progression [7,8,9].

Moreover, mutations in pancreatic cancer driver genes and pathways are closely intertwined with metabolic alterations. For instance, KRAS, a key PDAC driver gene, is involved in mediating glucose metabolism, autophagy, the reprogramming of de novo lipogenesis, and amino acid metabolism in PDAC cells [10].

Recently, the differential expression of genes associated with glycolysis and cholesterol synthesis has been found to predict survival in pancreatic cancer. In addition, heme metabolism and autophagy have emerged as key dependencies in this tumor [11,12].

Altered metabolism can modulate chemoresistance in several cancers, including PDAC [13,14,15].

For instance, a metabolic profiling study found that markers of amino acid metabolism could distinguish between gemcitabine-resistant and gemcitabine-sensitive PDAC cells [16]. Therefore, preclinical studies and clinical trials are investigating mechanisms to target metabolic dependencies for therapeutic purposes in PDAC [7,17,18].

### 1.3. Long Non-Coding RNA Functions and Their Dysregulation in Cancer

Long non-coding RNA LncRNAs are a group of transcripts of 200 nucleotides or longer, which have no or minimal protein-coding potential. Initially considered “junk” RNA, lncRNAs are now known to play a crucial role in regulating the human genome at several levels. Similar to protein-coding genes, lncRNA-expressing loci include a promoter, as well as multiple exons, and can be alternatively spliced. This class of molecules comprises RNAs transcribed either by polymerase I, polymerase II, or polymerase III. Some lncRNA loci are located in intergenic regions, whereas other lncRNAs are transcribed antisense to coding genes or by the alternative splicing of existing genes. Indeed, in around 17% of coding genes, the longest transcript is non-coding [19]. A subset of lncRNAs, called long interspersed RNA (LincRNA), derive from intronic regions. As opposed to coding genes, whose number does not significantly differ across species, the number of lncRNA is positively associated with organism complexity. More than 100,000 lncRNA loci have been identified to date; most of them express several isoforms, as the majority of non-coding exons undergo alternative splicing. Despite having a less conserved DNA sequence compared to protein-coding genes, lncRNA loci tend to have a conserved promoter exon structure and splice junctions. As opposed to most protein-coding genes, lncRNA transcription tend to be less ubiquitous; in fact, the expression of the majority of the known lncRNAs is tissue-specific [20,21,22].

Since their discovery, lncRNAs have emerged as master regulators of gene expression, with multiple functions and mechanisms of action. One of these is the interference with miRNA-mediated post-transcriptional regulation. Specifically, lncRNAs can act as “miRNA sponges”, as they can bind multiple miRNAs, thereby hampering their function. This is one of the mechanisms underpinning the interplay of large networks of different ncRNA classes with competing activities, known as competing endogenous RNAs (ceRNA) [23].

Specific lncRNAs can bind DNA, forming RNA–DNA hybrids such as R loops, or can directly interact with proteins through specific protein binding loops. For instance, the lncRNA telomeric repeat-containing RNA (TERRA), transcribed from telomeric DNA, contributes to telomere maintenance and telomere elongation by forming R-loops at telomeric ends and by directly binding shelterin complex proteins, such as TRF2 [24,25]. Some LncRNAs are involved in transcriptional regulation, mainly via gene silencing multiple neighboring genes (such as XIST) and the regulation of alternative splicing. Direct interaction with DNA is one of the mechanisms by which lncRNAs control chromatin remodeling. However, several lncRNAs can also participate in the regulation of the chromatin structure through an interaction with epigenetic modulators, such as Polycomb repressive complex 1 and 2 and HOX proteins, among others. Similar to miRNA sponging, some LncRNAs act as decoys for proteins by directly binding them and reducing their intracellular levels. LncRNAs can also act as scaffolds, and are emerging as a necessary component for the creation of nuclear condensates such as speckles and paraspeckles [26,27,28].

Moreover, it has been proposed that lncRNAs may also can act as enhancers, bringing transcription factors next to target gene promoters [22].

**Figure 1:**
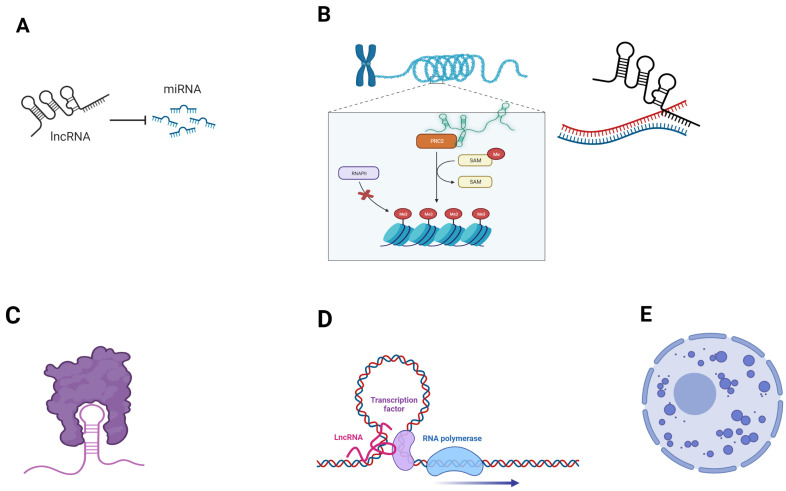
Examples of mechanisms by which lncRNAs exert their regulatory functions. (**A**) lncRNAs can act as miRNA sponges, sequestering them and impeding their regulatory functions on mRNAs; (**B**) chromatin remodeling and transcriptional regulation. LncRNAs can regulate chromatin architecture by interacting with epigenetic effectors, such as histone modificators (left). LncRNA:DNA binding is a mechanism of gene silencing (right); (**C**) protein sequestration by lncRNA; (**D**) lncRNAs may act as enhancers, bringing transcription factors close to transcription initiation site. (**E**) lncRNA scaffolding properties contribute to the formation of nuclear condensates. Created with BioRender.com (accessed on 5 June 2023).

LncRNA’s subcellular localization is consistent with their function. For instance, chromatin remodeling is mediated by lncRNAs located in the nucleus, whereas cytoplasmic lncRNAs are involved in transcriptional and post-transcriptional regulation, as well as in multiple signaling pathways. Moreover, specific lncRNAs that are located in the mitochondria (transcribed either from nuclear DNA or mitochondrial DNA) contribute to mitochondrial homeostasis [29]. An overview of the main lncRNA regulatory functions is reported in Figure 1.

Given the widespread and multifaceted role of lncRNAs in the regulation of biological functions, it comes as no surprise that lncRNA dysregulation is implicated in cancer [30,31,32].

Moreover, several lncRNAs contribute to metabolic reprogramming in cancer cells, mainly due to post-translational modifications of key metabolic players, such as HIF-1α and the c-Myc oncogene, as well as other cancer-associated proteins and pathways [33].

The role of lncRNAs in PDAC development and progression has been largely explored. Several LncRNAs are differentially expressed in PDAC, with different roles in tumorigenesis and progression depending on their downstream targets. The loss of the imprinting of H19, a paternally imprinted lncRNA, was suggested to play a role in pancreatic carcinogenesis more than two decades ago [34].

Additional studies confirmed that the upregulating H19 in human pancreatic cancer cells promotes tumor progression and EMT [35,36,37].

Among the lncRNAs that are overexpressed in PDAC is LINC01133, which is excreted in PDAC-derived exosomes, and contributes to PDAC progression through the upregulation of the Wnt/β-catenin pathway [38].

In addition to the overexpression of cancer-promoting lncRNAs, the downregulation of lncRNAs with tumor suppressive functions, such as FLVCR1-AS1, MIR600HG, and GAS8-AS1, can contribute to PDAC progression [39,40,41].

### 1.4. Diagnostic, Prognostic and Therapeutic Potential of LncRNAs—State of the Art, Advances and Caveats

The possibility of detecting cancer-associated lncRNAs in the serum of affected patients makes them potentially easily measurable cancer biomarkers. Multiple PDAC-associated lncRNAs have already been proposed as diagnostic and/or prognostic biomarkers, as well as predictors of response to treatment [42].

Recent studies, for instance, suggested that the expression of LINC00162 and ABHD11-AS1 could be used to detect early pancreatic cancer [43,44].

Moreover, multiple lncRNAs, including the extensively studied HOTTIP, HOTAIR, and PVT1, have been implicated in gemcitabine resistance, whereas others, like CASC2, AB209630, and GAS5, seem to improve responses to gemcitabine [45].

LncRNAs’ potential astherapeutic targets are currently being studied in multiple fields, and several mechanisms are used to inhibit lncRNA activity. Among these, RNA interference with small interfering RNA (siRNA) can silence and induce the post-transcriptional degradation of target lncRNAs. Antisense onligonucloeotides have been successfully used to exert a post-transcriptional silencing on lncRNAs derived from antisense genes, preventing them from cis-regulating neighboring genes. Moreover, aptamers are promising tools that could interfere with lncRNA–protein interaction. However, the current data are preliminary and derive from preclinical models, and the level of success of lncRNA targeting in-vitro has not yet been met during in-vivo studies. Research on lncRNA targeting is hampered by the poor conservation of this class of RNA, and, thus, the choice of models is limited and should be carefully chosen (human cell-lines, patient-derived xenografts, patient-derived tumor organoids). Moreover, there are potential issues concerning the optimal delivery method, and the bioavailability of these molecules and their target specificity, which should be addressed before deciding to proceed to clinical research [45,46,47,48]. However, the cell-lineage specificity of lncRNAs makes them potentially excellent candidates for targeted therapy, and, thus, efforts are still ongoing to overcome the above-mentioned limitations.

### 1.5. Search Strategy and Selection Criteria

For this review, we focused on original articles that demonstrated the role of single lncRNAs or lncRNA signatures in pancreatic cancer through the modulation of metabolic pathways that are known to be dysregulated in this type of cancer. Given the tissue specificity (and possibly isoform specificity) of lncRNAs, we only considered lncRNAs whose effect on specific metabolic pathways was demonstrated in PDAC, and did not include those described altered in PDAC, but for which experimental evidence of metabolic functions was observed in other tumors. No filter was applied concerning publication date.

## 2. Hypoxia-Responsive lncRNA

PDAC is characterized by an extensive desmoplastic stroma which, together with rapid cellular proliferation and insufficient vascularization, results in a highly hypoxic environment. This, in turn, contributes to the formation of a low-immune microenvironment. It has been demonstrated that, under inadequate oxygen supply conditions, cancer cells produce specific hypoxia-induced molecules. This metabolic reprogramming heavily relies on the activation of HIF-1, a major regulator of cellular adaptation to hypoxia [49].

Considering the role of hypoxia in PDAC, antagonization of the hypoxic microenvironment is being studied for PDAC treatment [14]. For instance, a phase 2 clinical study (NCT04141995) is studying the use of digoxin (a known cardiac glycoside used to treat heart arrythmias) as a modulator of the hypoxic microenvironment in combination with adjuvant chemotherapy in patients with resectable PDAC [50].

There is a growing body of literature on hypoxia-induced lncRNA, as well as lncRNAs that promote the expression of HIF-1. Indeed, exosomes secreted by hypoxic tumors, including pancreatic cancer cells, contain a cargo of differentially expressed ncRNAs compared to normal tissue, including several lncRNAs [51]. Hypoxia-induced lncRNAs promote glycolysis, tumor progression, and resistance to gemcitabine, as described below.

### 2.1. MTA2TR

The MTA2 transcriptional regulator lncRNA (MTA2TR) is enriched in PDAC samples compared to normal tissue, and has been inversely associated with poor overall survival in PDAC patients. In PDAC cell cultures, this lncRNA promotes the transcription of metastasis-associated protein 2 (MTA2), thereby promoting acetylation and, thus, the stabilization of HIF-1α, which would otherwise be degraded under normoxic conditions. This, in turn, results in an increase in HIF-1α transcriptional activity, and in a positive feedback loop between the latter and MTA2, possibly contributing to PDAC tumorigenesis and progression [52].

### 2.2. PVT1

Plasmacytoma Variant Translocation 1 (PVT1) has been positively associated with stage and negatively associated with prognosis in pancreatic cancer [53]. Similar to MTA2TR, PVT1 forms a positive feedback loop with HIF1-α, with both proteins stabilizing each other and promoting cell proliferation and metastatic potential. This loop is apparently relevant only in normoxic conditions as, during hypoxia, HIF1-α expression is not reduced following PVT1 siRNA transfection in PDAC cells, as occurs when oxygen supplies are sufficient [54].

### 2.3. HIF1A-AS1

HIF1A Antisense RNA 1 (HIF1A-AS1) is a lncRNA with antiangiogenic properties located on the long arm of chromosome 14, on the antisense strand of HIF-1. As demonstrated in endothelial cells, HIF1A-AS1 can suppress the expression of its target genes, including the pro-angiogenetic genes ADM and EPHA2, by directly binding double-strand DNA (dsDNA), thereby forming dsDNA/RNA triplexes [55]. HIF1-AS1, which is transcriptionally regulated by HIF-1α, promotes HIF-1 α protein translation in a positive feedback loop that has been implicated in the emergence of glycolysis-mediated gemcitabine resistance in PDAC cells [56].

### 2.4. LncRNA-CF129145.1

Another lncRNA, lncRNA-CF129145.1, suppresses cell proliferation in pancreatic cancer cultures and animal models by indirectly inhibiting p53-mediated FOXC2 transcription through p53 degradation. During hypoxia, lncRNA-CF129145.1 expression in PDAC cancer cells is suppressed by the HIF-1α/HDAC complex [57].

### 2.5. LncRNA-BX111887, ZEB-1AS1 and NR2F1-AS1

LncRNA-BX111887 (BX111), whose expression is induced by HIF-1α, promotes epithelial to mesenchymal transition through a positive cis-transcriptional regulation of the adjacent gene ZEB1, through YB-1 recruitment to its transcription site [58]. Antisense to ZEB1, ZEB-1AS1 is another lncRNA which is frequently overexpressed in PDAC cells. ZEB-1AS1 promotes PDAC proliferation and invasion due to a positive activation loop with HIF-1α. Indeed, ZEB-1AS1, whose expression is induced by HIF-1α during hypoxia, promotes ZEB-1-mediated HIF-1α protein stabilization [59]. In the case of NR2F1-AS1, another hypoxia-induced lncRNA, PDAC cell proliferation, invasion, and migration results from the indirect activation of the AKT/mTOR pathway, mediated by a positive cis-regulation of the NR2F1 transcription factor by NR2F1-AS1 [60].

### 2.6. RPL13AP23-201

The lncRNA previously described as ENST00000480739, now relabeled RPL13AP23-201 [61], has been found to be under expressed in PDAC compared to adjacent normal tissue, and its levels negatively correlate with nodal status and overall survival. In vitro and in vivo assays have shown that this lncRNA inhibits the migration and invasion of PDAC cells, whereas no effect on cell proliferation, death, or cell cycle regulation was observed. These assays also suggest that RPL13AP23-201 acts as an oncosuppressor by indirectly downregulating HIF-1α [62].

### 2.7. FEZF1-AS1

Another lncRNA, FEZF1-AS1, promotes cell proliferation and invasion by sponging two miRNAs, miR-142 and miR-133a, thereby removing the block on HIF-1α and EGFR expression. Similar to RPL13AP23-201, the effects of FEZF1-AS1 on PDAC cell proliferation through the modulation of HIF-1α appear to occur only in hypoxic environments [63]. The promotion of HIF-1α activity through miRNA sponging (miR-411-3p) is also a mechanism which has been observed for PCED1B-AS1, a lncRNA implicated in cell proliferation and epithelial-mesenchymal transition (EMT) in PDAC [64].

### 2.8. NORAD, LSAMP-AS1

NORAD, one of the lncRNAs whose expression is induced following hypoxic stimulation, has been shown to promote EMT in PDAC cell lines, as well as in animal models [65]. Moreover, from the analysis of expression of 200 hypoxia-associated genes, another lncRNA, LSAMP-AS1, emerged as one of the main players of a ceRNA network that regulated hypoxia, and its expression was found to be inversely associated with PDAC prognosis [66].

### 2.9. Linc-ROR

Specific PDAC-derived lncRNAs can also be excreted and modulate cancerogenesis by inducing modifications in other cell types in the tumor microenvironment. Long intergenic non-coding ROR (linc-ROR), for instance, is enriched within PDAC cell-derived exosomes and promotes the dedifferentiation of adipocytes, with a subsequent increase in PDAC cell proliferation and invasiveness through the upregulation of the HIF1α/ZEB1 signaling pathway [67].

### 2.10. UCA1

Another lncRNA found in PDAC exosomes released under hypoxia is UCA1, which promotes angiogenesis by modulating the miR-96-5p/AMOTL2/ERK1/2 signaling pathway [68]. Interestingly, UCA1 has also been found to be overexpressed in exosomes secreted by hypoxia-activated pancreatic stellate cells, and has been found to confer metastatic potential and gemcitabine resistance to neighboring PDAC cells [69].

ZNFTR hypoxia can also suppress the production of lncRNAs with tumor suppressive functions. ZNF24 Transcription Regulator (ZNFTR, also known as ZNF24TR), for instance, has the ability to impair cell proliferation and invasion by promoting the expression of ZNF24, a transcriptional repressor of VEGF. However, ZNFTR is downregulated in PDAC cells, and an underlying mechanism appears to be the deacetylation of ZNFTR promoter by the HIF1-α/HDAC1 complex [70].

## 3. Autophagy-Related LncRNA

The term autophagy (macroautophagy or macropinocytosis) describes the mechanisms by which intracellular material such as proteins and organelles are scavenged and included on intracellular vesicles, and degraded in the lysosomes in order to be recycled [71]. By removing and recycling damaged intracellular molecules such as proteins, autophagy prevents long term tissue degeneration, simultaneously providing nutrients independently of external sources. Autophagy levels can increase following cell starvation, which can occur, for instance, during hypoxic conditions. Moreover, the inhibition of mTOR signaling promotes autophagy. The upregulation of autophagy is a common oncogenic mechanism. However, the role of autophagy differs depending on the nature of the cells involved. In fact, according to experimental reports, autophagy appears to be protective against the neoplastic transformation of nonmalignant cells, but it boosts the progression of advanced cancer. Moreover, whether autophagy exerts anti-oncogenic or pro-oncogenic functions may be due to the type of stimulus that has induced autophagy and/or on the type of substrates that are being preferentially degraded [72]. Dysregulated autophagy has also been implicated in tumoral immune escape, although the underlying mechanisms are not completely elucidated, and data on the role of autophagy in tumor immunogenicity are conflicting [71].

Autophagy inhibitors are being studied for cancer treatment. However, research on these agents is complicated by the need to find the balance between treatment efficacy and the risk of toxicity, especially neurotoxicity, given the key role of autophagy in cell homeostasis [73]. Clinical trials are nevertheless ongoing to assess the effectiveness of autophagy-targeting agents on several types of malignancies [72,74]. In pancreatic cancer, increased autophagy has emerged as one of the main metabolic dependencies. Clinical trials investigating therapy with autophagy inhibiting agents rely mainly on hydroxychloroquine (HCQ), whose tolerability profile is well known, as it is a repurposed drug that is already used in the clinic. Phase 2 clinical trials have failed to demonstrate a benefit of (HCQ) in combination with gemcitabine or gemcitabine/nab-paclitaxel on advanced PDAC patients. However, a greater pathological response, as well as biochemical evidence of autophagy reduction and increased tumor immune infiltrate, were observed when the combination HCQ + gemcitabine/nab-paclitaxel was administered in the neoadjuvant setting [74,75]. Given the potential of autophagy targeting in PDAC, there is an urgent need for biomarkers that could aid in the selection of patients who could most benefit from these treatments. The identification of specific targetable molecules, such as lncRNA, expressed mainly in the tumor, may help to develop therapies with a high target specificity, in order to maximize the efficacy and minimize the systemic toxicity. Moreover, mounting evidence points to a pivotal role of cancer stem cells (CSC) in pancreatic carcinogenesis, and to their contribution to PDAC reprogramming [7]. Intriguingly, both enhanced autophagy and lncRNA altered expression have been shown to promote stemness in PDAC cells [76].

### 3.1. MALAT1/NEAT2

A series of specific PDAC-associated lncRNAs have been implicated in autophagy modulation. One of these is metastasis-associated lung adenocarcinoma transcript 1 (MALAT1)/noncoding nuclear-enriched abundant transcript 2 (NEAT2), a lncRNA that is overexpressed in several malignancies. MALAT-1 levels are higher in PDAC cells compared to normal pancreatic tissue, and its levels have been positively correlated with stage and negatively associated with patients’ survival [77,78]. Considering that MALAT-1 has been associated with an increased expression of autophagy-related proteins, and that MALAT-1 silencing results in a downregulation of these proteins both in vitro and in vivo, the role of this lncRNA in PDAC is likely due, at least in part, to autophagy [79].

### 3.2. PVT1

PDAC-associated lncRNAs can also play a role in chemotherapy resistance through the upregulation of autophagy. PVT1, for instance, in addition to its links to hypoxia, has also been implicated in gemcitabine resistance in PDAC through mechanisms involving other pathways. Indeed, a recent study suggested that PVT1 suppresses gemcitabine activity in PDAC cells by upregulating the Wnt-β-catenin signaling pathway, as well as autophagy-related pathways. The underlying mechanism appears to be a negative regulation of miR-619-5p by PVT1, which results in the increased expression of Pygo1 and ATG14, two proteins which are crucial for Wnt-β-catenin signaling and autophagy, respectively [80]. Moreover, PVT’s role in PDAC underscores the interconnection that exists between hypoxia mediators and autophagy pathways. Specifically, another mechanism by which PVT1 promotes autophagy is through the sponging of mir-143, which facilitates the HIF1-α-mediated upregulation of VMP1, a central player in the autophagy process [81].

### 3.3. SNHG14 and HCP5

Similar to PVT1, two other lncRNAs, SNHG14 and HCP5, can increase gemcitabine resistance by the upregulation of autophagy via a miRNA sponging mechanism [82,83].

### 3.4. Lnc-FSD2-31:1

Lnc-FSD2-31:1 expression is increased in tumor samples from long survivors (>5 years) compared to short survivor (<6 months) PDAC patients, and its role in prognosis may be due to the modulation of the peritumoral stromal microenvironment. Specifically, this lncRNA, excreted by PDAC cells via extracellular vesicles, increases autophagy in cancer-associated fibroblasts (CAFs) by removing the miR-4736 block on ATG7 expression, thereby hampering CAFs’ activation [84].

### 3.5. ANRIL/CDKN2B-AS1

ANRIL/CDKN2B-AS1, another cancer-associated lncRNA, appears to confer gemcitabine resistance by inhibiting autophagy. ANRIL has been found highly expressed in pancreatic cancer cells, together with HNGB1. Indeed, by a miRNA sponging mechanism, ANRIL removes the HMGB1 blockade by miR-181a and, in turn, HMGB1 promotes autophagy. In pancreatic cancer cell lines treated with gemcitabine, this results in increased cell proliferation in and gemcitabine resistance, which can be reverted by ANRIL silencing [85].

### 3.6. LINC01207 and LINC01133

As opposed to the above-mentioned lncRNAs, it has been postulated that LINC01207 promotes pancreatic cancer progression through the inhibition of autophagy. Indeed, LINC01207 is overexpressed in cancer cells. By negatively regulating miR-143-5 in human pancreatic cancer cells, this lncRNA increases the expression of AGR2, an endoplasmic reticulum protein implicated in multiple signaling pathways, which is overexpressed in several cancers and has been implicated in PDAC dissemination [86]. LINC01207 silencing, with subsequent increased levels of miR-143-5 and the reduced expression of AGR2, has been shown to inhibit cell growth and promote both apoptosis and autophagy in PDAC cells [87].

The LINC01133 lncRNA is known to be involved in PDAC cells’ proliferation and EMT. In addition to the above-mentioned oncogenic mechanisms (positive regulation of the Wnt/β-catenin pathway), LINC01133 can promote sponge miR-216a-5p, removing its inhibition on TPT1, a protein known to suppress autophagy by a positive regulation of both BECN1 and mTORC-1 signaling networks. Therefore, LINC01133 appears to promote cancerogenesis by suppressing autophagy, as demonstrated by increased levels of TPT1 levels and decreased levels of the autophagy markers LC3 in PDAC cells with LINC01133 overexpression, and by the drop of TPT1 levels following LINC01133 silencing [88,89].

These findings, apparently in conflict with the above-mentioned effects of other lncRNAs on autophagy in PC, possibly reflect the complexity of the autophagy process, which can act as a double-edged sword in cancer prevention and cancer promotion.

### 3.7. HOTAIR

Besides chemotherapy, autophagy-related lncRNAs may influence responses to other types of treatment. HOTAIR promotes autophagy in PDAC cells, as its expression is positively associated with that of ATG7, and could confer resistance to radiation therapy. In a study conducted on multiple pancreatic cancer cell lines, HOTAIR expression increased after irradiation. Interestingly, HOTAIR knockdown conferred radiosensitivity to these cells, which could be reverted by cell treatment with rapamycin, an autophagy-promoting molecule [90].

### 3.8. LncRNA Signatures

In addition to studies focused of the functional characterization of the role of single lncRNAs in PDAC, several gene expression studies have identified autophagy-associated lncRNA signatures and/or classificators which were associated with PDAC development and progression, as well as with prognosis and response to treatments. In some cases, signatures associated with the same outcome differed among studies, due, at least in part, to differences in the autophagy-associated gene sets selected for each study [91,92,93,94].

## 4. LncRNAs Implicated in Other Metabolic Pathways in PDAC

Although heme synthesis has emerged as a crucial metabolic dependency in PDAC, a role of heme-modulating lncRNAs in PDAC has not been experimentally demonstrated, to our knowledge. Conversely, a set of lncRNAs has been implicated in the dysregulation of lipid metabolism and amino acid metabolism in this tumor.

As previously mentioned, a subset pf PDAC exhibits a cholesterogenic gene expression signature [11]. Lipids are necessary for cell membrane stability, as well as for several biological processes that regulate cell growth and cell differentiation [95]. Normal tissues rely mainly on a dietary intake of lipids, whereas several types of cancer cells activate de novo lipogenesis to be independent of external sources. In fact, the inhibition of fatty acids and cholesterol synthesis results in impaired tumorigenesis, and is currently being studied as a therapeutic strategy for cancer treatment [96,97]. A phase 1 clinical trial, NCT04862260, is currently ongoing to assess the feasibility of cholesterol metabolism disruption with a triplet of cholesterol lowering drugs (evolocumab, atorvastatin, and ezetimibe) in combination with FOLFIRINOX in advanced PDAC [98].

In addition to lipid metabolism, amino acid metabolism is frequently altered in PDAC, especially that of glutamine. In fact, although glutamine is a non-essential amino acid, several tumors rely on glutamine metabolism for survival. In PDAC cells, glutamine metabolism rewiring is driven by the upregulation of the KRAS pathway [99]. Similar to lipid metabolism, glutamine dysregulation is a potential therapeutic target. Telaglenastat, a glutaminase inhibitor, is being investigated in clinical trials, alone or in combination with standard chemotherapy, for the treatment of several hematologic and solid malignancies. In PDAC, Glutamic-Oxaloacetic Transaminase 1 (GOT1) inhibition has shown promising results in preclinical studies, but further research is needed to assess the translatability of these results clinical trials [100,101,102,103].

### 4.1. SNHG16

Among the lncRNAs that are overexpressed in PDAC, SNHG16, which has an oncogenic activity in PDAC cells has been demonstrated to promote de novo lipogenesis by negatively regulating miR-195, thereby increasing the expression of its target SREBP2, a transcription factor involved in the activation genes which are involved in cholesterol synthesis [104,105].

### 4.2. ZFAS1

ZFAS1 has been implicated in cancer promotion through the dysregulation of liposynthesis in several malignancies. This lncRNA ZFAS1 is overexpressed in PDAC cells, where it increases the expression of 3-Hydroxy-3-Methylglutaryl-CoA Reductase (HMGCR), a rate-limiting enzyme in cholesterol synthesis, and of fatty acid synthase (FASN), which catalyzes the de novo biosynthesis of long-chain saturated fatty acids from acetyl-CoA and malonyl-CoA. ZFAS1 silencing in PDAC cells results in low levels of free fatty acids, cholesterol, triglycerides, and phospholipids, and in reduced PDAC proliferation and invasiveness [106].

### 4.3. XLOC_006390

The XLOC_006390 lncRNA is involved in PDAC progression through the upregulation of glutamate metabolism. XLOC_006390 overexpression results in increased intracellular levels of α-ketoglutarate (αKG), and has been associated with higher PDAC stage and shorter overall survival. By preventing c-Myc ubiquitination-mediated degradation, XLOC_006390 promotes the transcription of glutamate dehydrogenase 1 (GDH1), a gene that encodes a mitochondrial enzyme that converts glutamate into alpha-ketoglutarate and ammonia by oxidative deamination, and whose germline pathogenic variants cause a form of Familial Hyperinsulinism [107].

### 4.4. GSTM3TV2

In a study conducted on PDAC cell cultures, the GSTM3TV2 lncRNA was overexpressed in gemcitabine-resistant cells and was identified as a key player in a ceRNA network that has been implicated in modulating the response to gemcitabine. Specifically, by sponging the Let-7 miRNA, GSTM3TV2 promoted the expression of LAT2 and ORL1, two proteins involved in the uptake of neutral amino acids (such as glutamine) and in the reuptake of several molecules, including oxidized LDL [108,109,110].

**Figure 2:**
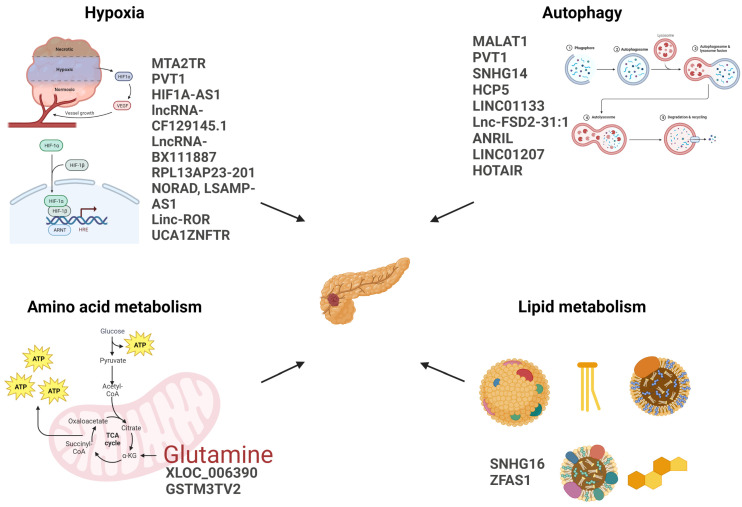
The figure shows the lncRNAs mentioned in this review, listed next to the metabolic pathways they affect. Created with BioRender.com (accessed on 01 June 2023).

An overview of lncRNAs involved in PDAC metabolic reprogramming is shown in Figure 2.

## 5. Conclusions and Future Directions

LncRNAs are an important component of the regulatory machinery, and contribute to the homeostasis of multiple cellular mechanisms. Energetic reprogramming, which is one of the main drivers of pancreatic cancer, is heavily influenced by lncRNA dysregulation, and metabolism-associated lncRNAs are not only promising predictive and/or prognostic biomarkers, but bear a therapeutic potential. In fact, metabolic dependencies are being studied as potential targets for novel therapies. Moreover, there is a large body of literature documenting the reversal of PDAC cell oncogenic potential by the direct modulation of specific lncRNAs, which, therefore, could be candidate targets for cancer treatment. Unfortunately, the reports on several lncRNAs are based on a single study, and this evidence needs additional confirmation to gain robustness. Therefore, considering that the therapeutic options are currently insufficient in PDAC, further research aimed at elucidating the role of metabolism-associated lncRNAs and at targeting them to address chemoresistance could be particularly relevant in the effort to improve the prognosis of PDAC patients.
